# A Hypothesis: Supplementation with Mushroom-Derived Active Compound Modulates Immunity and Increases Survival in Response to Influenza Virus (H1N1) Infection

**DOI:** 10.1093/ecam/neq037

**Published:** 2011-03-20

**Authors:** Han Chunchao, Jian-you Guo

**Affiliations:** ^1^School of Pharmacy, Shandong University of Traditional Chinese Medicine, Jinan 250355, China; ^2^Key Laboratory of Mental Health, Institute of Psychology, Chinese Academy of Sciences, Beijing 100101, China

## Abstract

We hypothesize that the mushroom-derived active compound may be a potential strategy for increasing survival in response to influenza virus (H1N1) infection through the stimulation of host innate immune response. The validity of the hypothesis can be tested by immune response to influenza infection as seen through survival percentage, virus clearance, weight loss, natural killer cell cytotoxicity, Tumor Necrosis Factor-**α** (TNF-**α**) and Interferon-gamma (IFN-**γ**) levels, lytic efficiency in the spleens of mice and inducible nitric oxide synthase mRNA expressions in RAW 264.7 murine macrophage cells. The hypothesis may improve people's quality of life, reduce the medical cost of our healthcare system and eliminate people's fears of influenza outbreak.

## 1. Introduction

The Pandemic (H1N1) 2009 has affected many countries and has already caused many human deaths. Fears are that further mutations in the virus could lead to a potentially more dangerous outbreak. Although vaccines are effective in preventing infection, vaccine coverage is incomplete and improvement in immunization is needed. The antiviral drugs such as amantadine and rimantadine now available for prophylaxis and treatment of influenza are limited by the rapid emergence of resistant virus mutants in the clinic.

As immune systems rely heavily on innate defenses, it has been suggested that stimulation of host innate immune response might provide prophylactic protection against influenza infection [1].

Considerable information and research exist on the identification of the biologically active components of medicinal mushrooms and on their use as therapies and immune system modulators. Mushrooms are of great interest due to the large number of biologically active compounds they contain. Some complexes from mushrooms are able to stimulate the non-specific immune system through the stimulation of the host's defence mechanism [2–16]. The immunomodulatory actions associated with mushrooms intake have been suggested as being due to a number of isolated fractions from mushrooms, including the *β*-d-glucans and other polysaccharides [17–19].

## 2. The Hypothesis

**Figure 1:**
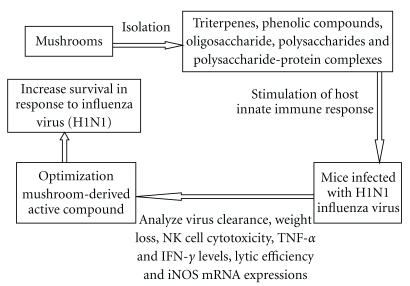
Diagram illustrating processing scheme of supplementation with MDAC to modulate immunity and increase survival in response to influenza virus (H1N1) infection.

Mushrooms need antiviral compounds to survive in their natural environment. It is therefore not surprising that antiviral compounds with more or less strong activities can be isolated from many mushrooms. Numerous studies have indicated that the antiviral effects of mushrooms result from the immunostimulating activity of polysaccharides or other complex molecules [17–22]. Active hexose correlated compound is a natural mushroom extract that has been reported to boost natural killer (NK) activity, improve survival and reduce the severity of H5N1 avian influenza in young mice [20]. The white button extracts readily stimulate macrophage production of TNF-*α* [21]. Ganodermadiol, lucidadiol and applanoxidic acid G isolated from the European Basidiomycete *Ganoderma pfeifferi* show antiviral activity against influenza virus type A and Herpes Simplex Virus type1 [22]. Taking all these together, we hypothesize that the mushroom-derived active compound (MDAC) will modulate immunity and increase survival in response to influenza virus (H1N1) infection (Figure 1). 

## 3. Testing the Hypothesis

### 3.1. Isolated MDAC from Mushrooms

*Ganoderma lucidum, Coprinus comatus, Grifola frondosa* and other species fungi of basidiomycetes are collected in China. Triterpenes, phenolic compounds, oligosaccharides, polysaccharides and polysaccharide-protein complexes are isolated, respectively. These mushroom-derived active compound (MDAC) are evaluated the effects of them on the immune response to influenza infection.

### 3.2. Influenza Virus Infection

Mice are anesthetized by intraperitoneal injection with Avertin and infected intranasally with the specified dose of H1N1 strain of mouse-adapted influenza A virus. The mice are monitored and weighed daily. This study should be performed in accordance with the Guidelines for Ethical Conduct in the Care and Use of Animals developed by the American Psychological Association. Care was taken to minimize discomfort, distress and pain to the animals.

### 3.3. Evaluation of Effects of MDAC on the Immune Response to Influenza Infection

The mice are supplemented with MDAC. Survival percentage, virus clearance, weight loss, NK cell cytotoxicity, TNF-*α* and IFN-*γ* levels, lytic efficiency in the spleens of mice and inducible nitric oxide synthase mRNA expressions in RAW 264.7 murine macrophage cells are analyzed.

## 4. Conclusions and Future Studies

In this article, we suggest that the MDAC may be a potential strategy to increase survival in response to influenza virus (H1N1) infection through the stimulation of host innate immune response. This hypothesis represents a completely novel area of study, which will lead to valuable treatments for and/or preventions of influenza virus (H1N1) infection. If the hypothesis is supported by further experimentation, it may improve people's quality of life, reduce the medical cost of our healthcare system and eliminate people's fears of influenza outbreak.

## Funding

Project of Shandong Province Higher Educational Science and Technology Program (J08LH62); Projects for Young Scientist from the Institute of Psychology in China (08CX043004), Project of National Natural Science Foundation of China (30800301).
